# Assessing PAH exposure during construction work of railroad switches using creosote-impregnated ties

**DOI:** 10.1093/annweh/wxag088

**Published:** 2026-10-15

**Authors:** Jessika Hagberg, Håkan Westberg, Bo Strandberg, Christian Lindh, Ing-Liss Bryngelsson, Pål Graff

**Affiliations:** Department of Occupational and Environmental Medicine, Faculty of Business, Science and Engineering, Örebro University, SE-701 82 Örebro, Sweden; Department of Occupational and Environmental Medicine, Faculty of Business, Science and Engineering, Örebro University, SE-701 82 Örebro, Sweden; Division of Occupational and Environmental Medicine, Department of Laboratory Medicine, Lund University, SE-221 00 Lund, Sweden; Department of Occupational and Environmental Medicine, Skåne University Hospital, SE- 223 81 Lund, Sweden; Division of Occupational and Environmental Medicine, Department of Laboratory Medicine, Lund University, SE-221 00 Lund, Sweden; Department of Occupational and Environmental Medicine, Örebro University Hospital, SE-701 16 Örebro, Sweden; Department of Chemical and Biological Work Environment, National Institute of Occupational Health, Po Box 5330 Majorstuen, 0304 Oslo, Norway

**Keywords:** pyrene, 1-hydroxypyrene, occupational exposure, dermal exposure, toxicokinetic model

## Abstract

The toxicological effects of creosote are primarily attributed to exposure to polycyclic aromatic hydrocarbons (PAHs) and related compounds. This study aimed to assess worker exposure by measuring PAHs in the breathing zone using full-shift air samples, urinary concentrations of 1-hydroxypyrene (1-HP), and dermal exposure through a tape stripping technique. Air sampling was conducted during a work shift on Day 4 (Thursday) of the work week, while urine and dermal samples were collected both before and after the shift on the same day. Additional urine samples were collected at the beginning of the week.

Measured concentrations of the 16 priority PAHs identified by the US EPA ranged from 120,000 to 280,000 ng/m^3^ in the breathing zones of workers handling creosote-impregnated ties. Pyrene concentrations on skin surfaces varied from 0.31 to 140 ng/cm^2^, with the highest concentrations found on palms and calves. Urinary concentrations of 1-HP increased over the course of the week, ranging from 1.9 to 17 µmol/mol creatinine, indicating substantial variation in individual exposure levels.

Workers constructing railroad switches with creosote-treated ties showed elevated urinary 1-HP concentrations during the work week, with most exceeding the British biological monitoring guidance value by Day 4. Dermal uptake was identified as the dominant exposure route using a physiologically based toxicokinetic model. Furthermore, pyrene was detected on skin areas covered by protective clothing, indicating that PAHs can penetrate fabric and that secondary exposure from contaminated work clothes is likely. These findings underscore the need for improved preventive measures to reduce dermal exposure.

What’s Important About This Paper This study provides quantitative evidence of substantial occupational exposure to polycyclic aromatic hydrocarbons (PAHs) among workers handling creosote-treated materials. By combining air monitoring, biomarker analysis, and dermal exposure assessment, the findings demonstrate that dermal absorption constitutes the primary exposure pathway, even through protective clothing. The observed exceedance of biological reference values for urinary 1-hydroxypyrene underscores the necessity for enhanced preventive strategies and improved protective measures to mitigate dermal uptake and secondary contamination from work garments.

## Introduction

Coal tar creosote, or creosote, is a by-product of the carbonization of coal to produce coke and has been used as a wood preservative since the late 19th century. Creosote is a mixture of many different compounds, such as polycyclic aromatic hydrocarbons (PAHs), phenols, and cresols (ATSDR 2024). Exposure to PAHs is associated with an increased risk of several cancers, including skin, lung, and bladder cancer (Boffetta et al. 1997; Bukowska et al. 2022; Barbosa et al. 2023). The International Agency for Research on Cancer (IARC 2010) has determined that coal tar creosote is carcinogenic to humans, and as it may pose a risk towards humans and wildlife, the use of wood preservatives containing creosote must be authorized for use in the European Union (EU). In Sweden, biocides containing creosote must be approved by the Swedish Chemicals Agency before use. Although its use is strictly regulated, industrial impregnation of railway ties and electricity and telephone poles is still permitted.

The toxicological effects of creosote originate from exposure to PAHs and related compounds. Occupational exposure may result from inhalation of creosote vapour and aerosol fractions or through skin contact. In creosote workers, dermal uptake is considered to be the most important route of exposure to PAHs (Jongeneelen 2001). Measurement of 1-hydroxypyrene (1-HP) in urine is a common method for biological monitoring of occupational exposure to PAHs (Jongeneelen et al. 1988; Elovaara et al. 1995; Louro et al. 2022). Urinary levels of 1-HP are a result of both dermal exposure and inhalation of pyrene. Dermal uptake can be a major contributor to total body dose in occupational settings, often exceeding 50% and reaching up to 90% among coke-oven and creosote-impregnation workers (Vanrooij et al. 1993a; Quinlan et al. 1995). To better understand exposure dynamics, Jongeneelen and ten Berge (2011) developed a physiologically based toxicokinetic model to predict urinary concentrations of 1-HP. This model, validated on coke and electrode paste workers, was shown to satisfactorily predict 1-HP excretion (Jongeneelen and ten Berge 2012). Using this model, it is possible to estimate the relative contributions of dermal and inhalation exposure to pyrene based on measured urinary 1-HP concentrations.

This study was conducted indoors in a facility where railroad switches are assembled with concrete or creosote impregnated wood ties. The aim of the study was to assess workers´ exposure to PAHs by (i) measuring 16 PAHs designated as high priority pollutants by the US EPA (US EPA PAH 16) in workers’ breathing zones from whole-shift samples, (ii) assessing workers’ internal exposure by measuring 1-HP in urine samples, (iii) examining dermal exposure to pyrene using a tape stripping technique, and (iv) using a toxicokinetic model to evaluate to what extent different exposure pathways (inhalation and dermal uptake) contribute to workers’ urinary concentrations of 1-HP, and thereby their internal exposure to PAHs.

## Materials and methods

### Study design

Urinary concentrations of 1-HP were used to monitor the internal (total) exposure to PAHs during the study week. To assess exposure to PAHs through inhalation of vapours and aerosols, PAHs were measured in air using both stationary and personal sampling. Stationary measurements were performed during Thursday work shifts at 5 different locations in the assembly hall of the facility and at other locations, such as the hallway (during 1 work shift), changing room (both work shifts) and in a detached building (1 shift). Nine workers handling creosote impregnated ties and 2 workers handling concrete ties wore personal samplers to measure PAHs in the breathing zone during Thursday work shifts. To assess dermal exposure, a tape stripping method was applied. Dermal samples were collected from the palms of workers and one of the controls before they started working but after they had changed into work clothes. After a work shift, dermal samples were collected from the palm, back of hand, forehead, and inside and outside of the right calf. Sampling of the 2 sides of the calf was used to investigate whether trousers provided any protection because the outside of workers’ trousers often had visible stains of creosote. This occurred when workers used their legs as they corrected the position of ties or supported themselves during certain tasks. During the study week, 600 m of creosote impregnated ties were used in the production of railroad switches. The week before, 200 m of creosote impregnated ties were used.

### Study group

Thirteen workers at a production plant of railroad switches using creosote-impregnated ties were included in the study. Four office employees were recruited as a small control group. All the workers had similar tasks, although they differed during the week. During the study week in January 2016, all the workers were in direct contact with creosote impregnated ties at some time. During the 2 work shifts on Thursday, 11 of the workers worked in direct contact with creosote-treated ties, whereas 2 of the workers worked solely with concrete ties. In the control group, 2 persons worked in the same building as the assembly hall and the other 2 worked in an adjacent building. These 2 persons working in the same building as the assembly hall changed into work clothes prior to their work shift and spent some time (up to 30 min) in the assembly hall in their roles as quality technician and group leader.

All study participants were men. Written informed consent was obtained from all participants and the Regional Ethics Committee in Uppsala, Sweden approved this study (Dnr 2016/049).

### Sampling

Urine samples were collected in prewashed polyethylene bottles during Monday morning (first void), during Thursday morning (first void), and after the end of the working shift during Thursday afternoon/evening depending on which work shift the workers belonged to. Air samples were actively sampled by drawing air at 2 L/min using a portable pump (SKC AirChek 5000, SKC Inc., Eighty Four, Pennsylvania, United States) through a sampling module consisting of 2 compartments: a closed phased cassette with a 2 µm pore size PTFE filter (Teflo, Pall Corporation, Port Washington, New York, United States) to determine particulate-associated PAHs, followed by a glass tube containing XAD-2 (SKC, Dorset, UK) to determine gas-phase PAHs. Air samples were collected during work shifts on Thursday. Dermal exposure was assessed on Thursday using a tape stripping method slightly modified from Kammer et al. (2011). Precut pieces (3 × 5 cm) of Fixomull tape (BSN Medical GmbH&Co, Hamburg, Germany) were applied to the skin with clean tweezers and left in place for 1 min. The tape was then removed with tweezers and placed in scintillation vials prefilled with 3 mL acetonitrile. A single tape was applied to each skin area, and the tweezer was rinsed with acetonitrile between each skin area sampled. Field blanks were also collected for air and dermal tape samples. Urine and dermal tape samples were kept at −20 °C until analysis.

### Chemical analyses

Urinary 1-HP was quantified using LC–MS/MS, while PAHs in air and dermal tape samples were analysed by GC–MS following validated protocols with rigorous quality control. Full details of analytical procedures, recovery rates, and quality assurance are provided in the Supplementary material and previously published methods (Kammer et al. 2011; Jorgensen et al. 2013; Alhamdow et al. 2017). The limit of detection (LOD) was determined using blank samples and was 0.060 ng/m^3^ for pyrene in air, 0.23 ng/cm^2^ for pyrene on dermal tape samples, and 0.050 ng/mL for 1-HP in urine.

### Modelling of 1-HP in urine

The physiologically based toxicokinetic model published by Jongeneelen and ten Berge (2011) was used based on measured air concentrations of pyrene in the breathing zone of the study participants as well as each individual’s dermal exposure to pyrene measured by tape stripping. In the toxicokinetic model, the duration of daily exposure was set to 8 h and the workers were assumed to use no respiratory protection. The area of exposed skin was estimated using values for the skin area of different body parts from Hoang (1992). Hands, half of the forearm, face, and one-quarter of the lower legs were considered exposed, resulting in an estimated exposed skin area of approximately 2,600 cm^2^. When comparing the predicted 1-HP levels from the model with measured urinary concentrations, the measured value of creatinine for the corresponding urine sample was used in the calculations. The toxicokinetic parameters for pyrene were predefined in the toxicokinetic model (see Table S1). To evaluate the contribution of the different exposure routes to the predicted urinary concentrations the model was tested under the following scenarios: only air exposure, only skin exposure, and combined skin and air exposure.

### Questionnaire and work diary

All study participants answered a questionnaire containing questions about gender, age, education, employment history, chronic disease, history of disease (cancer and cardiovascular disease), dietary habits, smoking habits, passive smoking, use of snus (oral smokeless tobacco product placed behind the upper lip), leisure activities related to PAH exposure, creosote-related work tasks (drilling, sawing, welding, transportation, etc.) and routines for wearing and changing protective clothing, including gloves, trousers, and short- or long-sleeve shirts, during the last 2 weeks. Summaries of the data for age, period of employment, and tobacco habits are shown in Table S2 and use of personal protective equipment in Table S3.

All study participants provided an additional detailed report of their different work tasks during Monday to Thursday in a work diary.

### Statistics

Descriptive statistics, including arithmetic mean, median, min and max, were used to evaluate the workers’ exposure to PAHs. Values below the LOD were substituted with LOD/2 before calculating mean and median concentrations. Spearman’s rank correlation coefficient was calculated (*P* = 0.05, 2-tailed) to assess the relationship between measured and predicted urinary concentrations of 1-HP among exposed workers on Thursday morning, as the data were not normally distributed. Statistical analyses were performed using STATA 17.0 (StataCorp LLC, Texas, United States) and IBM SPSS Statistics version 27.0 (SPSS Inc., Chicago, Illinois, United States). Modelling of the urinary concentrations of 1-HP was conducted using Microsoft Excel 2013 (Microsoft, Washington, United States) (Jongeneelen and ten Berge 2011).

## Results

### PAHs in air

The median concentration of US EPA PAH 16 (hereafter referred to as PAHs) in the breathing zones of 9 workers handling creosote-impregnated ties was 230,000 ng/m^3^ (mean: 220,000 ng/m^3^) (Table 1). The individual concentrations ranged from 160,000 to 280,000 ng/m^3^. The median and mean concentrations for the 2 workers handling concrete ties during the Thursday shifts were both 100,000 ng/m^3^, and their individual concentrations varied between 86,000 and 120,000 ng/m^3^. Stationary measurements were performed at 5 locations in the assembly hall during the morning shift and at 4 locations during the afternoon shift. Median and mean PAH concentrations of 200,000 ng/m^3^ and 240,000 ng/m^3^, respectively, were observed (Table 2). However, the median concentration of PAHs was lower (190,000 ng/m^3^) during the morning shift compared to the afternoon shift (340,000 ng/m^3^). Stationary air measurements were also conducted in the hallway connecting the assembly hall with offices and the lunchroom and in the changing room as well as in an adjacent building, showing median concentrations of 16,000 and 340 ng/m^3^, respectively (Table 2). The predominant PAHs in air were volatile 2 and 3 ring PAHs, such as naphthalene, fluorene, and phenanthrene. The particle-bound PAHs were at low concentrations and reflected more typical urban background levels of these PAHs (Strandberg et al. 2023).

**Table 1:** Mean, median, minimum, and maximum concentrations of US EPA PAH 16 in air (ng/m^3^) during personal sampling (full work shift).

Analyte	Personal measurement of PAH when working with impregnated ties (*n* = 9)			Personal measurement of PAH when working with concrete ties (*n* = 2)		
	Mean	Median	Min to Max	Mean	Median	Min to Max
Naphthalene	62,000	63,000	32,000 to 92,000	30,000	30,000	25,000 to 35,000
Acenaphthylene	450	450	310 to 560	190	190	160 to 210
Acenaphthene	110,000	120,000	110,000 to 120,000	52,000	52,000	43,000 to 61,000
Fluorene	22,000	24,000	9,000 to 28,000	10,000	10,000	870 to 11,000
Phenanthrene	21,000	22,000	16,000 to 25,000	9,300	9,300	8,500 to 10,000
Anthracene	430	440	300 to 560	160	160	130 to 190
Fluoranthene	470	450	250 to 840	210	210	180 to 250
Pyrene	190	180	97 to 390	77	77	65 to 89
Benzo[a]anthracene	0.87	0.70	0.17 to 3.2	0.23	0.23	3.5 to 1.1
Chrysene	1.3	1.1	0.55 to 3.6	0.65	0.62	0.43 to 0.88
Benzo(b)fluoranthene	0.41	0.41	0.34 to 0.51	0.36	0.36	0.30 to 0.42
Benzo(k)fluoranthene	0.14	0.12	0.08 to 0.30	0.11	0.11	0.084 to 0.14
Benzo(a)pyrene	0.095	0.086	0.05 to 0.17	0.049	0.049	0.016 to 0.081
Indeno-(1,2,3-c,d)pyrene	0.20	0.20	0.14 to 0.27	0.18	0.18	0.17 to 0.18
Dibenzo(a,h)anthracene	0.015	0.014	0.014 to 0.027	0.014	0.014	0.014 to 0.014
Benzo(g,h,i)perylene	0.16	0.13	0.094 to 0.33	0.11	0.11	0.094 to 0.13
**Sum US EPA PAH 16**	**220,000**	**230,000**	**160,000 to 280,000**	**100,000**	**100,000**	**86,000 to 120,000**

**Table 2:** Mean and median concentrations of US EPA PAH 16 in air (ng/m^3^) during stationary sampling (full work shift).

Analyte	Stationary measurement of PAH in workshop (*n* = 9)			Stationary measurement of PAH in hallway and changing room (*n* = 3)			Stationary measurement in adjacent building (*n* = 1)
	Mean	Median	Min to Max	Mean	Median	Min to Max	
Naphthalene	70,000	53,000	37,000 to 140,000	1,800	2,200	710 to 2,400	90
Acenaphthylene	530	410	240 to 1,200	24	24	18 to 30	1.3
Acenaphthene	120,000	99,000	47,000 to 260,000	7,200	7,700	5,900 to 8,100	140
Fluorene	26,000	21,000	12,000 to 55,000	2,600	2,400	2,200 to 3,000	40
Phenanthrene	20,000	17,000	9,800 to 41,000	3,300	3,400	3,100 to 3,500	68
Anthracene	350	280	120 to 760	47	46	52 to 55	0.53
Fluoranthene	230	200	110 to 530	55	49	35 to 81	1.3
Pyrene	90	79	38 to 210	20	17	13 to 30	0.93
Benzo[a]anthracene	0.24	0.24	0.068 to 0.50	0.047	0.036	0.013 to 0.093	0.013
Chrysene	0.67	0.64	0.35 to 1.1	0.30	0.30	0.24 to 0.37	0.26
Benzo(b)fluoranthene	0.40	0.40	0.29 to 0.68	0.27	0.27	0.25 to 0.28	0.19
Benzo(k)fluoranthene	0.099	0.091	0.069 to 0.15	0.067	0.062	0.057 to 0.083	0.062
Benzo(a)pyrene	0.070	0.063	0.036 to 0.12	0.036	0.036	0.034 to 0.039	0.016
Indeno-(1,2,3-c,d)pyrene	0.24	0.23	0.12 to 0.47	0.14	0.14	0.095 to 0.18	0.082
Dibenzo(a,h)anthracene	0.023	0.014	0.014 to 0.53	0.014	0.014	0.014 to 0.014	0.014
Benzo(g,h,i)perylene	0.19	0.14	0.11 to 0.53	0.06	0.06	0.040 to 0.084	0.041
**Sum US EPA PAH 16**	**240,000**	**200,000**	**110,000 to 500,000**	**15,000**	**16,000**	**12,000 to 17,000**	**340**

### Dermal exposure

Prior to the start of the work shift, but after changing into work clothes, dermal tape samples were taken from the palms of exposed workers and one of the controls. The median concentration of pyrene for workers prior to the work shift was 2.3 ng/cm^2^ and for one of the controls 2.7 ng/cm^2^ (Fig. 1, Table S5). After a work shift, dermal tape samples were taken from 5 different skin surfaces, ie the palm, back of hand, forehead, inside and outside of the right calf. Median concentrations of pyrene on the palm, back of hand, forehead, inside, and outside of the right calf were 21, 8.5, 6.1, 27, and 16 ng/cm^2^, respectively (Table S5). Individual concentrations of pyrene on the different skin surfaces varied between 0.31 and 140 ng/cm^2^ (Table S5). The highest median concentrations were detected on the calf and palm. Among the controls (*n* = 4), the median pyrene concentrations on the palm, back of hand, forehead, inside calf, and outside calf were 1.1, 0.76, 0.12, 0.49, and 0.46 ng/cm^2^, respectively (Table S5).

**Figure 1:**
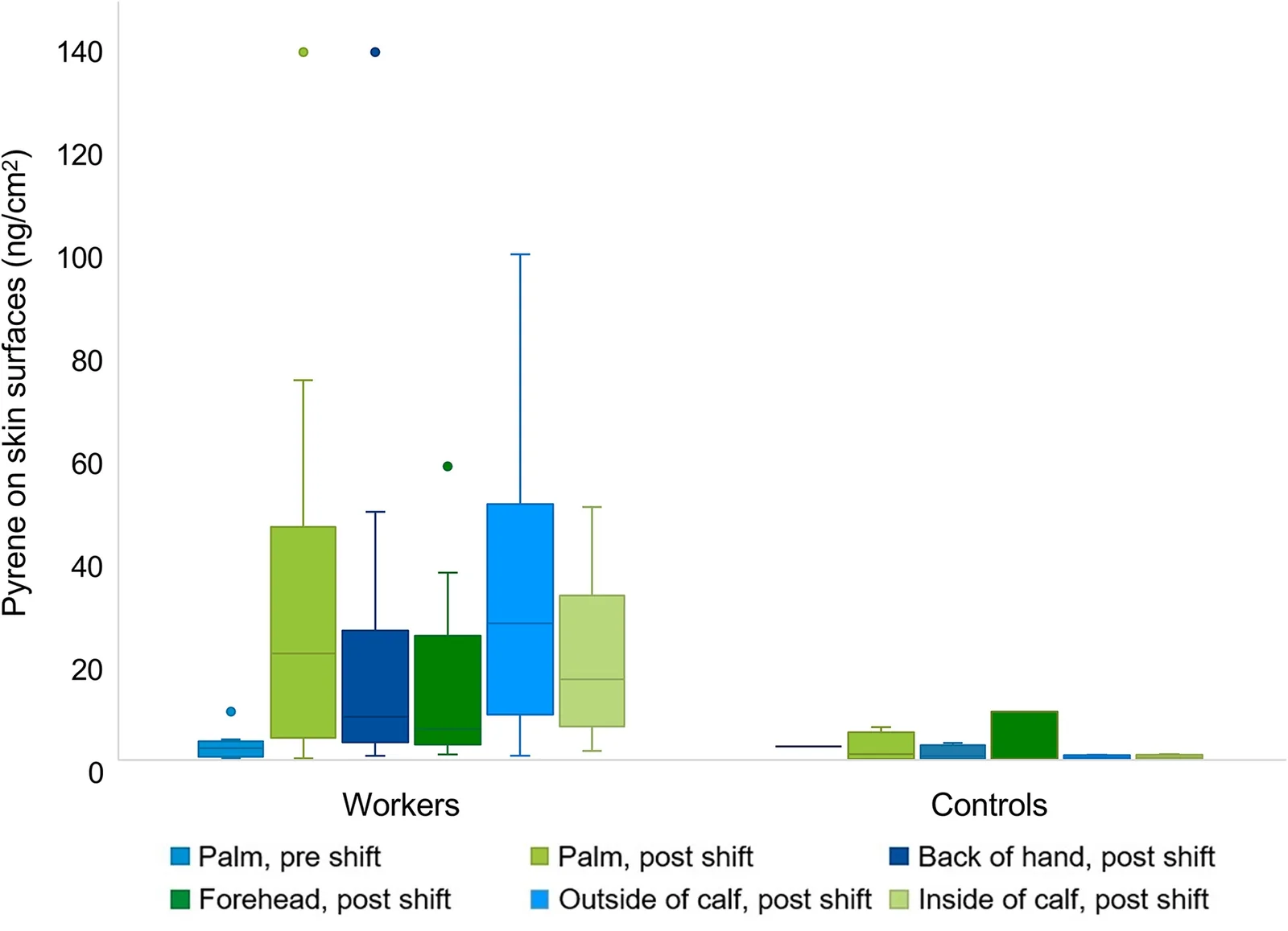
Boxplot of pyrene concentrations (ng/cm^2^) on different skin surfaces: palm, back of hand, forehead, and inside and outside of right calf. Boxes represent the interquartile range (IQR; 25th to 75th percentiles), with the median shown as a horizontal line. Whiskers extend to the most extreme observations within 1.5 × IQR from the lower and upper quartiles. Observations beyond the whiskers are shown individually and are considered statistical outliers according to this boxplot definition. All individual values, including statistical outliers, were retained in the descriptive statistics and modelling analyses.

### Internal exposure

The internal dose of PAH exposure was assessed by measuring urinary excretion of 1-HP. The first void was analysed for all study participants during Monday and Thursday, except for worker Q, who did not provide any urine samples (Table S6). After the Thursday work shift, a final urinary sample was collected. The median concentration of 1-HP in the exposed workers was 1.4 µmol/mol creatinine on Monday morning, compared to a median concentration of 0.19 µmol/mol creatinine detected in urine samples from the controls (Fig. 2, Table S6). In the exposed workers, the median concentration of 1-HP increased during the week and reached 5.0 and 4.6 µmol/mol creatinine in preshift and postshift samples collected on Thursday, respectively (Fig. 2). Among the controls, urinary concentrations increased slightly to 0.41 and 0.42 µmol/mol creatinine in the Thursday preshift and postshift samples, respectively. During Thursday, the individual concentrations of 1-HP varied between 1.9 and 17 µmol/mol creatinine (Table S6), indicating large variations in the workers’ exposure to pyrene and likely other PAHs.

**Figure 2:**
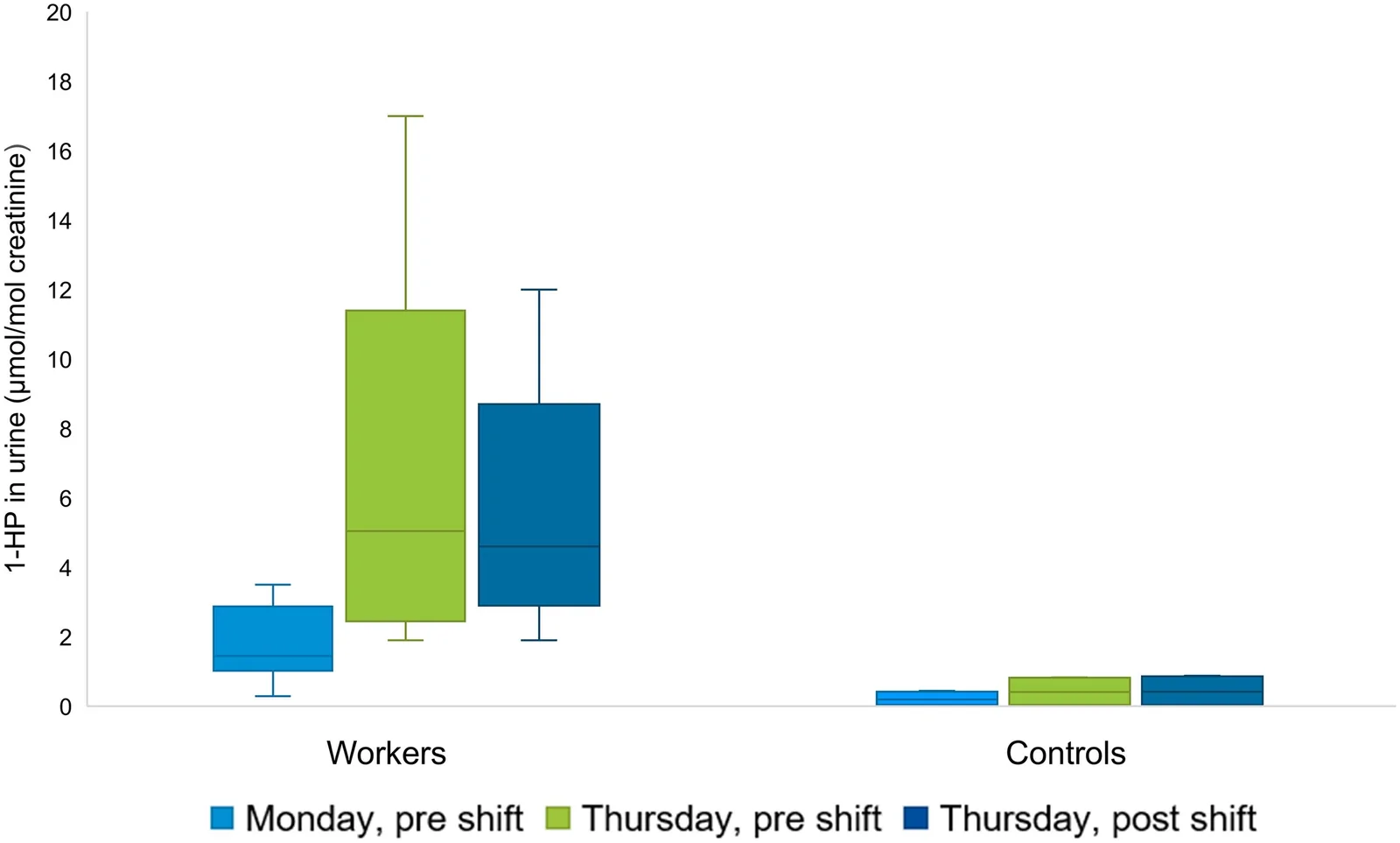
Boxplot of urinary concentrations of 1-hydroxypyrene (1-HP; µmol/mol creatinine) of exposed workers and controls. Boxes represent the interquartile range (IQR; 25th to 75th percentiles), with the median shown as a horizontal line. Whiskers extend to the most extreme observations within 1.5 × IQR from the lower and upper quartiles.

### Modelling of urinary concentrations of 1-HP

To predict the urinary concentrations of 1-HP (µmol/L), a toxicokinetic model published by Jongeneelen and ten Berge (2011) was used. Figure 3 illustrates the predicted urinary concentrations of 1-HP based on measured airborne and dermal pyrene exposure for 1 worker. Dermal exposure to pyrene in ng/cm^2^/hour was calculated as the difference between preshift dermal concentrations on the palm and the mean postshift concentration across the 5 sampled skin areas. The area of exposed skin was estimated to be 2,600 cm^2^ (Hoang 1992).

**Figure 3:**
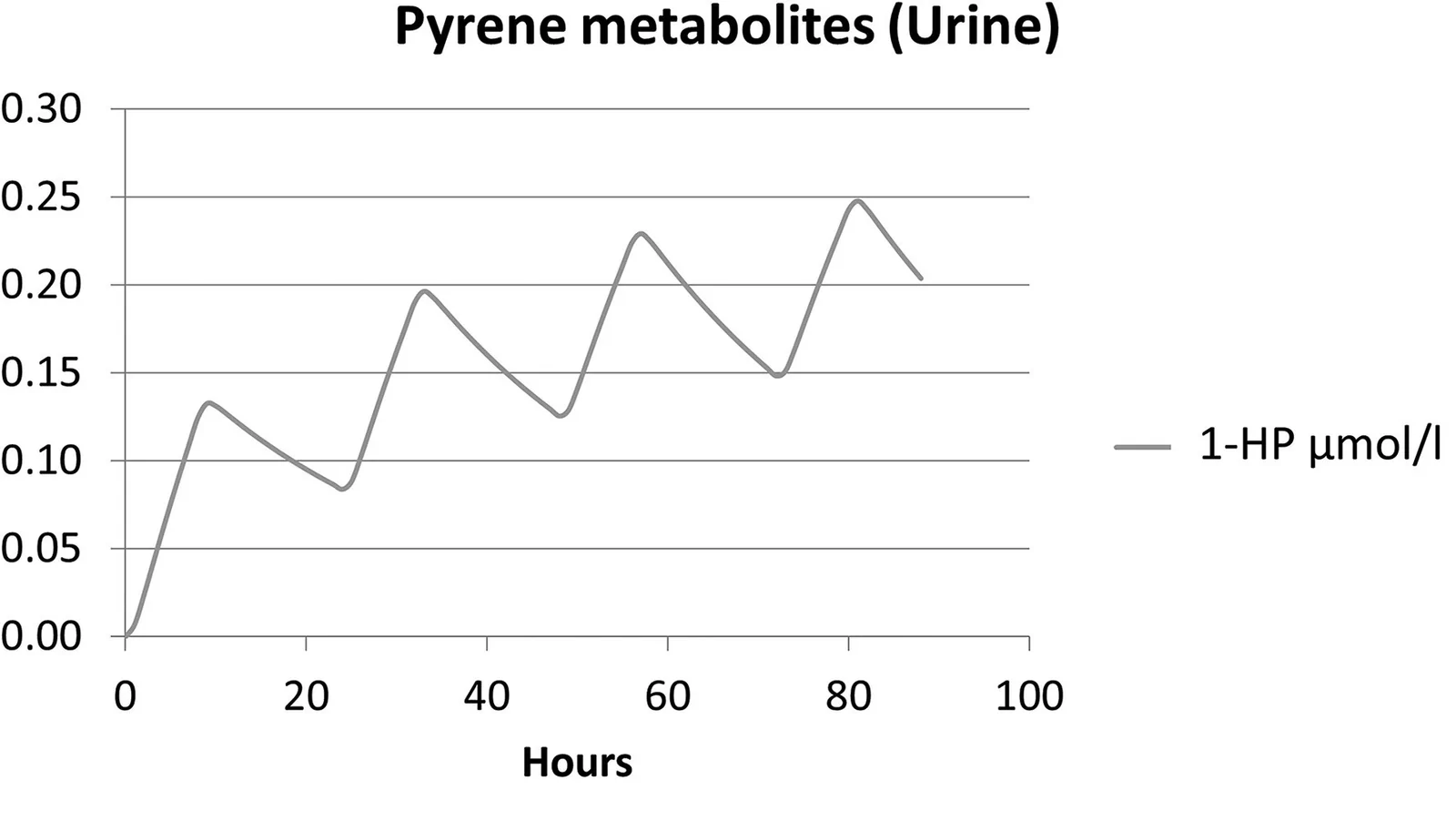
Illustration of predicted urinary levels of 1-hydroxypyrene (1-HP). The start of the timeline is set to the beginning of the work shift on Monday.

To evaluate the relative contribution of different exposure routes, the model was run separately using only air exposure, only dermal exposure, and combined dermal and air exposure. Dermal exposure had the greatest impact on the predicted urinary concentrations of 1-HP, accounting for an average of 95% of the predicted 1-HP concentrations obtained when both dermal and air exposure were included. In the subsequent calculations, results based on combined skin and air exposure were used. Figure 4 shows a scatterplot comparing the predicted urinary concentrations of 1-HP with the measured levels in urine samples collected on Thursday morning. To determine whether there was a correlation between the measured and predicted urinary values, Spearman’s rank correlation coefficient between the measured and predicted urinary values on Thursday morning was calculated. To improve the estimation of predicted urinary concentrations, each worker’s background level of 1-HP (Monday morning urine) was added to the predicted increase during the work week. The measured concentrations of creatinine for the corresponding sample were used when adjusting for creatinine. Spearman’s rank correlation coefficient between measured and predicted urinary 1-HP concentrations on Thursday morning was 0.76.

**Figure 4:**
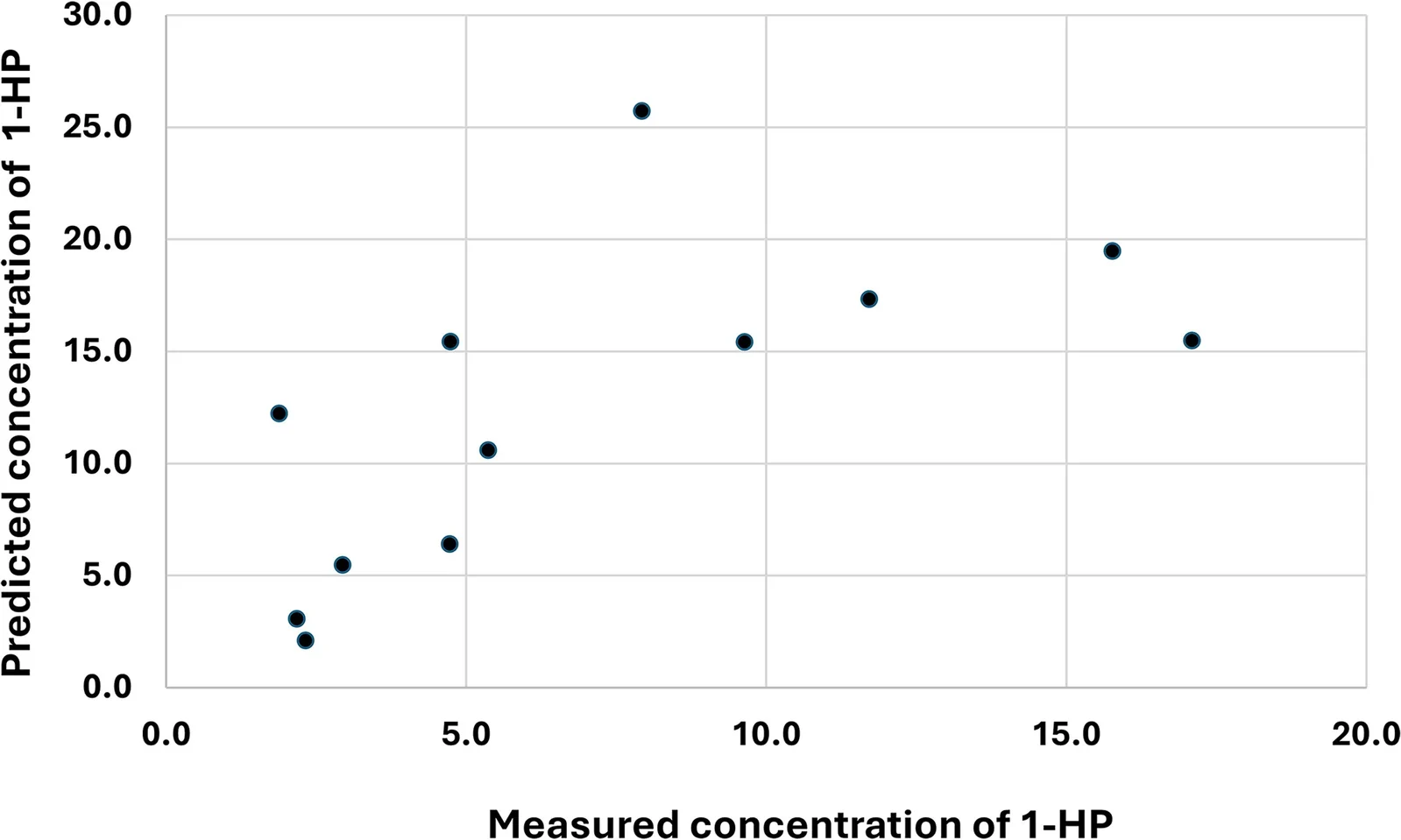
Scatter plot of measured and predicted urinary concentrations of 1-hydroxypyrene (1-HP) for urine samples collected before the work shift on Thursday. All concentrations are presented in µmol/mol creatinine. Urine 1-HP levels are normalized to creatinine for the corresponding samples. The skin surface area used for modelling dermal uptake was 2,600 cm^2^.

## Discussion

### Exposure via air

Median PAH concentrations in air were 200,000 ng/m^3^ for stationary sampling and 230,000 ng/m^3^ for personal sampling (Tables 1 and 2). Stationary measurements were notably higher during the afternoon shift (340,000 ng/m^3^) compared to the morning shift (190,000 ng/m^3^), indicating that the exposure increased later in the day. Concentrations varied across locations in the assembly hall and among the exposed workers during the sampling day. PAH concentrations were highest in the vicinity of the creosote-treated ties and were also elevated in the hallway connecting the assembly hall with offices and the lunchroom, and in the changing room (Table 2). The median concentrations of PAHs were similar in the hallway and the changing room even though the changing room was situated on the second floor. Since the workers only changed their working clothes weekly, this suggests contamination from the work clothes. In an adjacent building with offices and without creosote-related production, the median air concentration was 340 ng/m^3^ (Table 2).

For pyrene, median concentrations were 180 ng/m^3^ for personal measurements and 79 ng/m^3^ for stationary measurements in the assembly hall (Tables 1 and 2). These concentrations are lower than previously reported for personal measurements during work with creosote. Elovaara et al. (2006) reported mean pyrene concentrations of 530 to 1,390 ng/m^3^, whereas Vanrooij et al. (1993b) measured concentrations of 300 to 3,000 ng/m^3^. For comparison with other Swedish occupational settings, pyrene concentrations ranging from 80 to 450 ng/m^3^ have been reported in an alloy industry, and from 570 to 7,300 ng/m^3^ in a coke plant (Bohlin et al. 2010). The exposure levels in the alloy industry were similar to those observed in the present study, whereas concentrations in the coke plant were considerably higher.

For 2 of the PAHs, ie naphthalene and benzo[a]pyrene, the Swedish Work Environment Authority has established occupational exposure limits (OELs) of 50 and 0.002 mg/m^3^, respectively. In the present study, all the measured air concentrations of both compounds were at least 2 orders of magnitude below these limits. However, ongoing EU initiatives indicate that future regulations are likely to entail a substantially lower acceptable exposure level than those currently in force for benzo[a]pyrene (EU 2025). An OEL of 70 ng/m^3^ has been proposed aiming to reduce occupational cancer risk rather than as a threshold below which no risk exists. The median benzo[a]pyrene exposure observed in this study (86 ng/m^3^) exceeds the proposed risk-based EU OEL of 70 ng/m^3^. This highlights the distinction between existing regulatory limit values and exposure levels proposed on the basis of cancer prevention considerations, underlining the continued occupational health relevance of such exposures.

Although benzo[a]pyrene is widely regarded as a suitable marker for assessing PAH-related health risks, the carcinogenic activity of the PAH mixture in air was further characterized by estimating its cumulative carcinogenic potential (Petry et al. 1996). Benzo[a]pyrene equivalents (BaP-eq) were calculated according to national guidelines from the Swedish Environmental Protection Agency and international standards (Larsen and Larsen 1988; Boström et al. 2002). This approach accounts for differences in carcinogenic potency among individual PAHs by converting their concentrations into BaP equivalents using established potency equivalency factors (Larsen and Larsen 1988; Nisbet and Lagoy 1992). Accordingly, the measured PAH concentrations in the breathing zone were expressed as BaP-eq. The combined carcinogenic contribution of the 16 PAHs corresponded to 500 ng/m^3^ BaP-eq in air samples with the highest PAH concentrations. This value is approximately 4 times lower than the current Swedish OEL of 2,000 ng/m^3^ for benzo[a]pyrene, suggesting that inhalation exposure to PAHs may not be a primary concern using the existing OEL. However, these levels remain concerning in light of the proposed lower OEL aimed at reducing occupational cancer risk. It is noteworthy, however, that PAHs in the gas phase accounted for more than 99% of the total toxicity. Overall, these findings warrant continued efforts to reduce exposure and strengthen risk management for PAHs and creosote in occupational settings.

### Dermal exposure of pyrene

There were substantial differences in the concentrations of pyrene on skin surfaces between the exposed and nonexposed (controls) workers (Fig. 1). Large differences were also observed between samples taken from the palms before and after the work shift. Among the exposed group, the highest median concentrations were detected on skin surfaces outside the calf (median: 27 ng/cm^2^), followed by the palms (median: 21 ng/cm^2^), and the inside of the calf (median: 16 ng/cm^2^; Fig. 1, Tables S4 and S5). For the 2 workers handling only concrete ties (workers A and Q), pyrene concentrations on skin surfaces were among the lowest observed in the exposed group, although they remained higher than the median levels in the control group for all skin surfaces except the palm of worker Q.

In the present study, a single tape was applied to each skin area, as Kammer et al. (2011) suggested that 1 tape strip is sufficient to obtain an estimate of dermal exposure. However, their experimental results demonstrated that the mean recovery of pyrene from the skin was 63.3% and 70.2% when 5 consecutive tape strips were applied after 0 and 30 min, respectively (Kammer et al. 2011). Furthermore, Kammer et al. (2011) reported that the first tape strip recovered 62.2% and 40.4% of the total amount obtained from 5 consecutive tape strips in skin samples from 5 volunteers and 5 chimney sweeps, respectively. These findings indicate that a single tape strip does not recover the entire amount of pyrene present on the skin. Consequently, the dermal pyrene levels measured in the present study are likely to represent an underestimation of the actual skin contamination. Dermal contamination is expected to vary with work tasks, contact patterns, and local exposure conditions. However, for modelling purposes, dermal exposure was assumed to be uniformly distributed across body sites, representing a simplifying assumption in the absence of site-specific exposure data.

Dermal exposure to PAHs, and pyrene in particular, has been studied previously for several occupational groups. Somewhat higher concentrations than in this study were detected among coke oven (0.1 to 50.9 ng/cm^2^; range of geometric means) and aluminium smelter (6.0 to 192.4 ng/cm^2^; range of geometric means) workers, and those involved in wood impregnation (0.7 to 109.9 ng/cm^2^; range of geometric means) and remediation work of creosote-contaminated soils (2.5 to 350 ng/cm^2^) (Vanrooij et al. 1992; Dor et al. 2000). Skin surfaces on the wrist and neck/face showed the highest dermal concentrations in these studies. Several of the cited studies employed a different method for assessing dermal exposure, namely skin-mounted pads. In a study by Vanrooij et al. (1994), the efficiency of skin-mounted pads was compared to a skin wipe technique, showing that the latter was 48% to 62% more effective. In the present study, tape stripping was used to assess dermal exposure. As no published comparisons exist between tape stripping, skin-mounted pads, and the skin wipe technique, direct comparisons of contaminant concentrations across skin surfaces should be interpreted with caution. However, comparative assessments of which skin areas were most exposed remain valid and informative.

Additionally, skin surfaces under protective clothing (ie in the groin) were found to have been exposed to relatively high pyrene concentrations (Vanrooij et al. 1992; Vanrooij et al. 1993b; Vanrooij et al. 1994). A similar observation was made in the present study, ie pyrene concentrations on the outer and inner side of the calf were comparable to concentrations detected on the palm (Fig. 1 and Tables S4 and S5) even though the calf was protected by work trousers throughout the working day. Concentrations reached up to 100 ng/cm^2^ on the outer calf (Table S5), similar to pyrene concentrations reported in aluminium smelter workers’ groin (up to 106 ng/cm^2^; Vanrooij et al. 1992). The highest individual concentrations were detected on the palm and back of the hand (140 ng/cm^2^). Although gloves were intended to be worn during all handling of creosote-impregnated ties and replaced daily, inconsistent use and re-wearing of contaminated gloves may have contributed to elevated concentrations observed on hands. In addition, prolonged use of contaminated gloves may result in occlusion and increased skin hydration, which may enhance dermal absorption of lipophilic compounds as PAHs, potentially contributing to increased internal exposure.

These findings indicate that creosote penetrated the protective clothing. As work clothes were only changed once per week (on Mondays), whereas gloves were replaced daily, contaminated garments may have served as a secondary exposure source over the course of the working week. Workers handling creosote-impregnated sleepers early in the week might be exposed to creosote later the same week via clothing even without direct contact with creosote-containing material. Two workers assigned to concrete ties on the sampling day (ie, workers A and Q in Table S5) showed low pyrene concentrations overall. However, concentrations on their calves were comparable to or slightly higher than those on their palms, suggesting limited secondary exposure from workwear. Given the small sample size, this observation should be interpreted with caution.

### Biological monitoring of 1-HP

The exposed workers had considerably higher urinary concentrations of 1-HP (median 5.1 µmol/mol creatinine) compared with the controls (median 0.41 µmol/mol creatinine) and nonoccupationally exposed controls from Sweden (median: 0.03 to 0.09 µmol/mol creatinine) (Jongeneelen 2001; Alhamdow et al. 2018). In addition, urinary concentrations among the exposed workers were significantly higher than the German biological reference value for 1-HP for nonsmokers (BAR; 0.3 µg/g creatinine, equivalent to 0.16 µmol/mol creatinine) (DFG 2025), confirming that the measured urinary levels of 1-HP are attributable to occupational exposure.

Among the exposed workers, median 1-HP concentrations in first void urine during Monday morning were 1.5 µmol/mol creatinine (Fig. 2, Table S6), suggesting similar exposure to pyrene during the previous week despite higher use of creosote impregnated ties during the study week, assuming a urinary half-life of approximately 20 h (Hansen et al. 2008). Internal exposure increased over the week, reaching 5.0 and 4.6 µmol/mol creatinine on Thursday morning and afternoon, respectively.

Postshift Thursday concentrations ranged between 2.7 and 12 µmol/mol creatinine for the exposed workers and the highest concentration (ie 17 µmol/mol creatinine) was detected in the Thursday preshift measurements. These concentrations are comparable but slightly lower than those reported in Finnish switch production using creosote-impregnated ties (mean 8.4; range 0.4 to 25.1 µmol/mol creatinine; Heikkila et al. 1995). Comparably, a German study monitoring exposure during the assembly of railroad sleepers at 3 plants between 2005 and 2017 reported median urinary 1-HP concentrations of 4.7 µmol/mol creatinine (Hebisch et al. 2020). Higher exposures have been documented during wood impregnation in Finland (27 to 122 µmol 1-HP/mol creatinine; Elovaara et al. 1995) and The Netherlands (40 and 85 µmol 1-HP/mol creatinine; Jongeneelen et al. 1988), whereas somewhat lower exposure levels of 1-HP (<0.05 to 33 µmol/mol creatinine) were reported among workers at a US facility where railroad ties were heat- and pressure-treated with creosote (Borak et al. 2002).

The slightly lower concentrations observed in this study compared to those in the impregnation industry are reasonable, as creosote exposure in such environments is likely more extensive than in switchgear manufacturing. In addition, current protective measures are possibly more effective than in the older studies. However, our results indicate that they are not sufficient. The concentrations measured in the present study are comparable to other occupational groups, such as coke plant workers (Vanrooij et al. 1993a) and aluminium smelters (Quinlan et al. 1995). However, pyrene and its metabolite are not carcinogenic in themselves, but are commonly used as a biomarker for exposure to other, more carcinogenic PAHs. The relationship between carcinogenic PAHs and pyrene may vary depending on the exposure media (ie soot, creosote; Neff et al. 2005). While urinary 1-HP is widely considered a suitable biomarker of overall PAH exposure across many occupational settings (Unwin et al. 2006), differences in PAH composition between source materials may limit the direct comparability of urinary 1-HP concentrations across workers exposed to different PAH sources and corresponding cancer risk estimates.

Urinary 1-HP concentrations were low for the control group, with a median concentration of 0.19 µmol/mol creatinine on Monday morning and rising to 0.41 and 0.42 by Thursday morning and afternoon, respectively (Table S6). Thursday concentrations were 10 times higher than those reported in nonoccupationally exposed populations (0.03 µmol/mol creatinine), suggesting low ongoing exposure during the working week. Two controls working in offices in the same building as the workshop were exposed to pyrene to some extent (Table S6), likely due to brief visits in the assembly hall as they had roles as supervisors and quality technicians. The urinary 1-HP concentrations for the other 2 controls who worked in a detached building ranged between 0.03 and 0.04 µmol/mol creatinine, confirming no occupational exposure.

There are no Swedish biological guidance values for 1-HP in urine, nor within the EU (Bolt et al. 2016). However, the British Health and Safety Executive recommend a biological monitoring guidance value (BMGV) of 4 µmol/mol creatinine (Unwin et al. 2006; Bolt et al. 2016). Postshift concentrations above the BMGV indicate increased occupational PAH exposure, requiring employers to take measures to reduce workers’ exposure. In this study, the majority of Thursday postshift urinary concentrations among exposed workers exceeded the BMGV. Since air measurements indicated low exposure through the workplace air, dermal uptake was suspected as the main exposure route. To test this, predicted urinary 1-HP concentrations based on dermal and air measurements were compared with observed concentrations.

### Modelling of urinary metabolites

According to the physiologically based toxicokinetic model applied, the urinary concentrations of 1-HP increased on each day in connection with exposure and decreased between work shifts. The model also predicted a gradual increase in urinary concentrations over the course of the work week (Fig. 3). A relatively strong Spearman’s correlation coefficient (rho = 0.76) was observed between measured urinary concentrations on Thursday morning and predicted urinary concentrations resulting from combined dermal and inhalation exposure. This correlation was observed despite skin exposure was evaluated on only 1 day during the work week and despite probable day-to-day variation in exposure, even though work tasks were relatively consistent. The observed correlation between the predicted and measured urinary concentrations also indicated that, for most workers, pyrene exposure probably originated from their occupation as discussed earlier. The predicted urinary post-shift levels diverged more from the measured concentrations (rho = 0.49). This discrepancy may be due to variability in exposure timing and intensity during the work shift, but it may also reflect a predominance of dermal exposure, which can result in delayed excretion of 1-HP (Sobus et al. 2009).

In addition, the toxicokinetic model allowed estimation of the contribution of air exposure versus dermal exposure to the measured urinary concentrations of 1-HP. By comparing urinary concentrations of 1-HP from skin exposure and air exposure independently, the contribution of the measured dermal exposure was found to explain 95% of the total measured urinary concentrations of 1-HP. This estimate aligns with the findings by Vanrooij et al. (1993b), who reported that up to 90% of the total occupational exposure among creosote-impregnation workers was attributable to dermal exposure, underscoring the critical role of dermal uptake (Jongeneelen 2001). Consequently, strategies to reduce workers’ exposure should prioritize minimizing dermal exposure and improving hygiene practices. This indicates that the model can be useful not only to predict excretion of metabolites, but also to evaluate exposure routes and thus possible preventive measures.

One limitation of the toxicokinetic model used in this study was the uncertainty in estimating the skin area relevant for dermal uptake. Although parts of the lower legs were covered by work trousers, pyrene was detected in both the outer and inner sides of the calves, indicating that contaminated clothing may have contributed to dermal exposure through transfer or penetration of the fabric. Therefore, one-quarter of the lower leg area was included in the estimation of exposed skin area. However, the true skin area contributing to uptake is uncertain, as exposure may have resulted from both direct contact with the impregnated ties and secondary transfer from contaminated clothing. The exposed area may also have varied between workers depending on glove use, work practices and transfer of contamination to the face by touching with contaminated hands or gloves. As the assumed exposed skin area affects the predicted urinary concentrations, the model was also tested using a reduced skin area of 1,000 cm^2^ (Fig. S2). The mean difference between the predicted and measured urinary 1-HP concentrations was 1.0 µmol/mol creatinine with a standard deviation (SD) of 4.0 when using an assumed skin area of 1,000 cm^2^, compared with 5.2 µmol/mol creatinine (SD 5.5) when using 2,600 cm^2^. This highlights the uncertainty involved when both exposure intensity and exposed surface area may vary between body sites and workers.

The predominance of dermal exposure, together with the finding that most workers exceeded the British BMGV for urinary 1-HP, underscores the need for measures to reduce dermal uptake. Although the study was conducted in 2016, the results remain relevant, as creosote-treated railway ties are still permitted in Sweden and in parts of the EU when technically necessary. Consequently, occupational exposure to creosote and PAHs continues to be a concern not only for railway switch construction workers, as studied here, but also for workers involved in the handling, installation, removal, and disposal of creosote-treated ties, as well as other occupations with dermal PAH exposure (eg asphalt workers, roofers, and chimney sweeps). Given the well-established association between PAH exposure and cancers of the skin, lung, and bladder (Boffetta et al. 1997; Barbosa et al. 2023), reducing dermal exposure is crucial. However, when introducing protective measures such as protective clothing, it is important to define appropriate laundering procedures to prevent contaminated workwear from becoming a secondary source of exposure (Stec et al. 2018).

## Conclusions

During construction of railroad switches made from creosote impregnated ties, workers exhibited elevated urinary concentrations of 1-HP by the fourth day of the work week. Most workers had urinary concentrations exceeding the British BMGV of 4 µmol/mol creatinine, indicating that measures are needed to reduce exposure. Dermal absorption was identified as the dominant route of exposure, contributing approximately 95% of the total uptake.

When assessing dermal exposure across different skin surfaces, the highest pyrene concentrations were detected on the outside and inside of the calves and on palms. Both areas were partially or fully covered by gloves and protective trousers throughout the workday. These findings suggest that pyrene, and likely other PAHs, can penetrate the fabric of gloves and trousers. Furthermore, as the same protective trousers are worn throughout the week, secondary exposure from contaminated work clothing appears likely. This observation suggests that reliance on protective clothing may provide a false sense of security, highlighting the need for improved protective strategies and hygiene measures.

Future research should focus on developing fabrics with greater resistance to PAH penetration and evaluating whether more frequent changes of protective clothing effectively reduce exposure. Another important area is to investigate the efficiency of current washing routines in removing nonpolar contaminants and to identify improvements where necessary.

Although measured air concentrations of naphthalene and benzo[a]pyrene were below current Swedish OELs, the observed benzo[a]pyrene levels exceeded the proposed risk-based EU OEL for cancer prevention. This highlights the need to assess occupational exposures not only against existing regulatory limits but also against emerging health-protective standards, and to continue preventive efforts to further reduce PAH exposure.

## Supplementary Material

wxag088_Supplementary_Data

## Data Availability

The data underlying this article are available in the article and in its online Supplementary material.

## References

[wxag088-B1] Agency for Toxic Substances and Disease Registry (ATSDR). 2024. Toxicological profile for creosote. Atlanta GA, U.S. Department of Health and Human Services, Public Health Service.

[wxag088-B2] Alhamdow A et al 2017. Early markers of cardiovascular disease are associated with occupational exposure to polycyclic aromatic hydrocarbons. Sci Rep. 7:Article 9426. 10.1038/s41598-017-09956-x. PMC5573323 28842704

[wxag088-B3] Alhamdow A et al 2018. DNA methylation of the cancer-related genes F2RL3 and AHRR is associated with occupational exposure to polycyclic aromatic hydrocarbons. Carcinogenesis. 39:869–878. 10.1093/carcin/bgy059. PMC6030939 29722794

[wxag088-B4] Barbosa F et al 2023. Polycyclic aromatic hydrocarbons (PAHs): updated aspects of their determination, kinetics in the human body, and toxicity. J Toxicol Environ Health-Part B-Crit Rev. 26:28–65. 10.1080/10937404.2022.2164390. 36617662

[wxag088-B5] Boffetta P, Jourenkova N, Gustavsson P. 1997. Cancer risk from occupational and environmental exposure to polycyclic aromatic hydrocarbons. Cancer Causes Control. 8:444–472. 10.1023/A:1018465507029. 9498904

[wxag088-B6] Bohlin P, Jones KC, Strandberg B. 2010. Field evaluation of polyurethane foam passive air samplers to assess airborne PAHs in occupational environments. Environ Sci Technol. 44:749–754. 10.1021/es902318g. 20000814

[wxag088-B7] Bolt H, Heederik D, Papameletiou D, Klein C. 2016. SCOEL/REC/404 Polycyclic aromatic hydrocarbon mixtures containing benzo[a]pyrene (PAH), Recommendation from the Scientific Committee on Occupational Exposure Limits. Publications Office of the European Union. 10.2767/977505.

[wxag088-B8] Borak J, Sirianni G, Cohen H, Chemerynski S Jongeneelen F. 2002. Biological versus ambient exposure monitoring of creosote facility workers. J Occup Environ Med. 44:310–319. 10.1097/00043764-200204000-00011. 11977417

[wxag088-B9] Boström CE et al 2002. Cancer risk assessment, indicators, and guidelines for polycyclic aromatic hydrocarbons in the ambient air. Environ Health Perspect. 110:451–488. 10.1289/ehp.110-1241197. PMC1241197 12060843

[wxag088-B10] Bukowska B, Mokra K, Michalowicz J. 2022. Benzo a pyrene-environmental occurrence, human exposure, and mechanisms of toxicity. Int J Mol Sci.;23:6348. 10.3390/ijms23116348. PMC9181839 35683027

[wxag088-B11] Deutsche Forschungsgemeinschaft (DFG) . 2025. List of MAK and BAT values 2025: Maximum concentrations at the workplace and assessment values in biological material. Permanent Senate Commission for the Investigation of Health Hazards of Chemical Compounds in the Work Area, Report 61. German Medical Science. 10.34865/mbwl_2025_eng.

[wxag088-B12] Dor F et al 2000. Feasibility of assessing dermal exposure to PAHs of workers on gaswork sites—the SOLEX study. Sci Total Environ. 263:47–55. 10.1016/S0048-9697(00)00665-3. 11194162

[wxag088-B13] Elovaara E, Heikkilä P, Pyy L, Mutanen P, Riihimäki V. 1995. Significance of dermal and respiratory uptake in creosote workers: exposure to polycyclic aromatic hydrocarbons and urinary excretion of 1-hydroxypyrene. Occup Environ Med. 52:196–203. 10.1136/oem.52.3.196. PMC1128187 7735394

[wxag088-B14] Elovaara E, Mikkola J, Mäkelä M, Paidanius B, Priha E. 2006. Assessment of soil remediation workers’ exposure to polycyclic aromatic hydrocarbons (PAH): biomonitoring of naphthols, phenanthrols, and 1-hydroxypyrene in urine. Toxicol Lett. 162:158–163. 10.1016/j.toxlet.2005.09.028. 16263227

[wxag088-B15] European Commission (EU) . 2025. Proposal for a directive of the European Parliament and of the Council amending Directive 2004/37/EC as regards the addition of substances and setting limit values in its Annexes I, III and IIIa. COM(2025) 418 final), 2025/0232 (COD). Brussels.

[wxag088-B16] Hansen Å, Mathiesen L, Pedersen M, Knudsen LE. 2008. Urinary 1-hydroxypyrene (1-HP) in environmental and occupational studies-A review. Int J Hyg Environ Health. 211:471–503. 10.1016/j.ijheh.2007.09.012. 18222724

[wxag088-B17] Hebisch R et al 2020. Inhalation and dermal exposure of workers during timber impregnation with creosote and subsequent processing of impregnated wood. Environ Res. 181:108877. 10.1016/j.envres.2019.108877. 31722805

[wxag088-B18] Heikkila P, Luotamo M, Pyy L, Riihimake V. 1995. Urinary 1-naphthol and 1-pyrenol as indicators of exposure to coal-tar products. Int Arch Occup Environ Health. 67:211–217. 10.1007/BF00626355. 7591181

[wxag088-B19] Hoang KT . 1992. Dermal exposure assessment: principles and applications (interim report EPA/600/8-91/011B). U.S. Environmental Protection Agency, Office of Health and Environmental Assessment, Exposure Assessment Group.

[wxag088-B20] International Agency for Research on Cancer (IARC) . 2010. Some Non-heterocyclic Polycyclic Aromatic Hydrocarbons and Some Related Exposures. IARC Monographs on the Evaluation of Carcinogenic Risks to Humans, Vol. 92, Lyon, France.

[wxag088-B21] Jongeneelen F, ten Berge W. 2011. A generic, cross-chemical predictive PBTK model with multiple entry routes running as application in MS Excel; design of the model and comparison of predictions with experimental results. Ann Occup Hyg. 55:841–864. 10.1093/annhyg/mer075. 21998005

[wxag088-B22] Jongeneelen F, ten Berge W. 2012. Simulation of urinary excretion of 1-hydroxypyrene in various scenarios of exposure to polycyclic aromatic hydrocarbons with a generic, cross-chemical predictive PBTK-model. Int Arch Occup Environ Health. 85:689–702. 10.1007/s00420-011-0713-9. 22038087

[wxag088-B23] Jongeneelen FJ . 2001. Benchmark guideline for urinary 1-hydroxypyrene as biomarker of occupational exposure to polycyclic aromatic hydrocarbons. Ann Occup Hyg. 45:3–13. 10.1016/S0003-4878(00)00009-0. 11137694

[wxag088-B24] Jongeneelen FJ, Bos RP, Henderson PT. 1988. Metabolites of polycyclic aromatic-hydrocarbons in urine of exposed workers. Toxicol Environ Chem. 16:295–307. 10.1080/02772248809357268.

[wxag088-B25] Jorgensen RB, Strandberg B, Sjaastad AK, Johansen A, Svendsen K. 2013. Simulated restaurant cook exposure to emissions of PAHs, mutagenic aldehydes, and particles from frying bacon. J Occup Enviro Hyg. 10:122–131. 10.1080/15459624.2012.755864. 23343415

[wxag088-B26] Kammer R, Tinnerberg H, Eriksson K. 2011. Evaluation of a tape-stripping technique for measuring dermal exposure to pyrene and benzo(a)pyrene. J Environ Monit. 13:2165–2171. 10.1039/c1em10245a. 21687840

[wxag088-B27] Larsen J, Larsen P. 1988. Chemical carcinogens. In: Hester R, Harrison R, editors. Air pollution and health. Cambridge UK, The Royal Society of Chemistry. p. 33–56.

[wxag088-B28] Louro H et al 2022. The use of human biomonitoring to assess occupational exposure to PAHs in Europe: a comprehensive review. Toxics. 10:0480. 10.3390/toxics10080480. PMC9414426 36006159

[wxag088-B29] Neff JM, Stout SA, Gunster DG. 2005. Ecological risk assessment of polycyclic aromatic hydrocarbons in sediments: identifying sources and ecological hazard. Integr Environ Assess Manag. 1:22–33. 10.1897/IEAM_2004a-016.1. 16637144

[wxag088-B30] Nisbet ICT, Lagoy PK. 1992. Toxic equivalency factors (TEFs) for polycyclic aromatic hydrocarbons (PAHs). Regul Toxicol Pharmacol. 16:290–300. 10.1016/0273-2300(92)90009-X. 1293646

[wxag088-B31] Petry T, Schmid P, Schlatter C. 1996. The use of toxic equivalency factors in assessing occupational and environmental health risk associated with exposure to airborne mixtures of polycyclic aromatic hydrocarbons (PAHs). Chemosphere. 32:639–648. 10.1016/0045-6535(95)00348-7. 8867146

[wxag088-B32] Quinlan R et al 1995. Polycyclic aromatic hydrocarbon exposure in coal-liquefaction workers—the value of urinary 1-hydroxypyrene excretion in the development of occupational hygiene control strategies. Ann Occup Hyg. 39:329–346. 10.1016/0003-4878(95)00004-X. 7793752

[wxag088-B33] Sobus J et al 2009. Comparing urinary biomarkers of airborne and dermal exposure to polycyclic aromatic compounds in asphalt-exposed workers. Ann Occup Hyg. 53:561–571. 10.1093/annhyg/mep042. PMC2723216 19602502

[wxag088-B34] Stec AA et al 2018. Occupational exposure to polycyclic aromatic hydrocarbons and elevated cancer incidence in firefighters. Sci Rep. 8:2476. 10.1038/s41598-018-20616-6. PMC5802792 29410452

[wxag088-B35] Strandberg B, Omelekhina Y, Klein M, Krais AM, Wierzbicka A. 2023. Particulate-bound polycyclic aromatic hydrocarbons (PAHs) and their nitro- and oxy-derivative compounds collected inside and outside occupied homes in southern Sweden. Polycycl Aromat Compd. 43:7399–7415. 10.1080/10406638.2022.2136218.

[wxag088-B36] Unwin J, Cocker J, Scobbie E, Chambers H. 2006. An assessment of occupational exposure to polycyclic aromatic hydrocarbons in the UK. Ann Occup Hyg. 50:395–403. 10.1093/annhyg/mel010. 16551675

[wxag088-B37] Vanrooij J et al 1992. Dermal exposure to polycyclic aromatic hydrocarbons among primary aluminium workers. Med Lav. 83:519–529. 1297067

[wxag088-B38] Vanrooij J, Maassen L, Bodelier-Bade MM, Jongeneelen FJ. 1994. Determination of skin contamination with exposure pads among workers exposed to polycyclic aromatic hydrocarbons. Appl Occup Environ Hyg. 9:693–699. 10.1080/1047322X.1994.10388396.

[wxag088-B39] Vanrooij JGM, Bodelierbade MM, Jongeneelen FJ. 1993a. Estimation of individual dermal and respiratory uptake of polycyclic aromatic-hydrocarbons in 12 coke-oven workers. Br J Ind Med. 50:623–632. 10.1136/oem.50.7.623. PMC1035498 8343423

[wxag088-B40] Vanrooij JGM, Vanlieshout EMA, Bodelierbade MM, Jongeneelen FJ. 1993b. Effect of the reduction of skin contamination on the internal dose of creosote workers exposed to polycyclic aromatic-hydrocarbons. Scand J Work Environ Health. 19:200–207. 10.5271/sjweh.1322. 8367698

