# Supplementary material for: Assessing PAH exposure during construction work of railroad switches using creosote-impregnated ties
Source: Ann Work Expo Health. 2026 Oct 15;70(8):wxag088. doi: 10.1093/annweh/wxag088 (PMC13671403; doi:10.1093/annweh/wxag088)
Supplement: Supplementary file 1 [file wxag088_supplementary_data.pdf]

SUPPLEMENTARY MATERIAL

ASSESSING PAH EXPOSURE DURING CONTRUCTION WORK OF RAILROAD SWITCHES USING CREOSOTE IMPREGNATED TIES

Jessika Hagberg[1*], Håkan Westberg[1], Bo Strandberg[2,3], Christian Lindh[2], Ing-Liss Bryngelsson[4], Pål Graff[5]

[1]Department of Occupational and Environmental Medicine, Faculty of Business, Science and Engineering, Örebro University, SE-701 82 Örebro, Sweden

[2]Division of Occupational and Environmental Medicine, Department of Laboratory Medicine, Lund University, SE-221 00 Lund, Sweden

[3]Department of Occupational and Environmental Medicine, Skåne University Hospital, SE-223 81, Lund, Sweden

[4]Department of Occupational and Environmental Medicine, Örebro University Hospital, SE-701 16 Örebro, Sweden

[5]National Institute of Occupational Health, Oslo, Norway

*Corresponding author: jessika.hagberg@oru.se

Tel: +46 196022461

List of tables and figure in supporting material

Table S1. Parameters used in the modelling of 1-HP in urine.

Table S2. Age, length of employment at current workplace and tobacco habits among the study participants.

Table S3. Use of personal protective equipment (PPE).

Table S4. Mean, median, min and max concentration of pyrene (ng/cm^2) on different skin areas before and after a work shift.

Table S5. Individual concentrations (ng/cm^2) of pyrene on different skin surfaces before and after a work shift. Samples were collected from exposed workers (*n*=13) and non-exposed controls (*n*=4).

Table S6. Individual concentrations of 1-hydroxypyrene in urine (µmol/mol creatinine) for exposed workers and controls during the sampling week.

Figure S1. Certificates: German External Quality Assessment Scheme.

Figure S2. Scatter plot of measured and predicted urinary concentrations of 1-hydroxypyrene (1-HP) for urine samples collected before the work shift on Thursday. All concentrations are presented in µmol/mol creatinine. Urine 1-HP levels are normalised to creatinine for the corresponding samples. The skin surface area used for modelling dermal uptake was 1000 cm^2.

Chemical analysis

To assess the total individual PAH exposure, the pyrene metabolite 1-HP was analysed using liquid chromatography coupled to tandem mass spectrometry (LC–MS/MS; QTRAP 5500, AB Sciex Framingham, MA, USA) at Lund University, Sweden. Details of the measurements have been published previously (Alhamdow et al. 2017). In brief, internal standard (IS) of 1-HP was added to the urine samples and hydrolysed using glucuronidase. All samples were prepared in duplicate, and the average concentration was further used. Blank samples and two quality control urine samples were analysed in every analytical batch. The laboratory participates in the G-EQUAS inter-laboratory exercise for the analysis of 1-HP coordinated by the University of Erlangen-Nuremberg, Germany, certificates are included in Figure S1. Concentrations of 1-HP in urine were normalized to the urinary concentration of creatinine (µmol/mol creatinine).

PAHs in air were analysed by gas chromatography mass spectrometry (GC-MS; Agilent 5975C connected to a 7890A MSD, Agilent Technologies, Inc., Santa Clara, CA, US). The filters were analysed for particulate associated PAHs and the XAD-2 sorbents were analysed for gas phase PAHs. These components were processed separately, and the reported air concentrations represent the sum of both phases. The extraction and clean up protocols are described in detail in Bramming Jørgensen et al. (2013). In brief, an IS mixture containing 16 deuterated U.S. Environmental Protection Agency (US EPA) priority PAHs was added to each sample. The samples were then extracted in 3 mL dichloromethane by means of a Sonica ultrasonic extractor (Soltec, Milan, Italy). Extracts were cleaned up through Pasteur pipettes filled with silica gel and then concentrated by purging with nitrogen to a final volume of 20-30 µL. Final extracts were injected onto a DB-5 capillary column (60 m x 0.32 mm I.D., 0.25 µm film thickness; J&W, Agilent). MS was performed in the electron impact (EI) ionization mode using selected ion monitoring (SIM). The method is assured with native mixtures of the 16 US EPA PAHs. Every target compound was quantified using the relative response factor to each native compound included in the mixture. The quality of the analysis was ensured with internal standard recoveries, field and lab blanks, and certified reference material (SRM 1649a urban dust) used as quality control (QC). Consistent recovery (60-120%) was obtained for the IS compounds. A somewhat lower recovery (ca 50%) was achieved for naphthalene that can be attributed to the higher volatility for this compound and thus slightly higher losses during sample work-up. The blanks were analysed in parallel with the samples. Some minor residues were found in the blanks, but in no case was the amount of any compound in any

blank greater than 15% of the amount found in any sample. All PAH results were corrected to the blanks. The limit of detection (LOD) was calculated as three times the standard deviation of the blanks. The measured concentrations of 12 PAHs in the QCs rarely deviated more than 30% from the certified concentrations. We judged these results to be acceptable.

To assess dermal exposure to PAHs, tape samples of the outermost surface of epidermis were analysed using GC-MS (Agilent 7890B coupled to a 5977A MSD, Agilent Technologies, Inc., Santa Clara, CA, US). The extraction and clean up protocols were slightly modified from Kammer et al. 2011. First, an IS mixture of 16 deuterated US EPA priority PAHs was added to each tape sample stored in separate scintillation vials containing acetonitrile. After 30 minutes, 5 mL of acetonitrile was added to each vial and the samples were extracted for 30 minutes using ultra sonication. Afterwards, the acetonitrile extracts were transferred to separate clean glass tubes, an additional aliquot of 5 mL acetonitrile was added to each sample in the scintillation vials and the extraction process was repeated. For each sample, the two acetonitrile extracts were combined and then evaporated to dryness using a vacuum centrifuge, thereafter the analytes were re-dissolved in *n*-hexane. The extracts were cleaned up over Florisil cartridges (500 mg/3 mL FL; Biotage) using solid phase extraction. The cartridges were rinsed with 6 mL *n*-hexane:dichloromethane (1:1) before the sample extracts were added. The extracts were eluted using 6 mL *n*-hexane:dichloromethane (1:1), then concentrated to a volume of approximately 25 μL and transferred to GC-vials to which an aliquot of recovery standard had been added. The extracts were allowed to concentrate under a gentle stream of nitrogen and then a surplus of toluene was added. The extracts were concentrated to a final volume of 30 μL. A volume of 2 μL of the final extract was injected onto a 30 m Select PAH column (0.25 mm I.D., 0.15 μm film thickness; J&W, Agilent). Recoveries of the IS compounds ranged from 45 to 70%. Laboratory blanks and field blanks were run in every batch of ten samples. Pyrene concentrations in blanks were low (less than 0.5% of the concentrations detected in samples) and no blank correction was performed.

Table S1. Parameters used in the modelling of 1-HP in urine.

Exposure model =	3. Normal man (light work)
Concentration parent compound (mg/m^3)	As air measurement
Time of start of airborne exposure (hours)	0
Duration of airborne exposure per day (hours/day)	8
Respiratory protection factor (=> 1)	1
Dermal protection factor (air tight clothing => 1)	10
Parameters of dermal exposure to parent compound	
Skin deposition pure substance (mg/cm^2/hour)	As measured on tape strips
Start of skin exposure (hours)	0
Duration of skin exposure per day (hours/day)	8
Skin temperature (centigrade)	25
Affected skin area (cm^2)	2600
Parameters of oral absorption	
Bolusdose to stomach of parent compound (mg/kg bwt)	0
Time of application (time in hours)	0
Absorption rate into intestinal tissue (1/hour)	3
Setting of simulation parameters	
Select human or experimental animal subject (1-14)	3
Length of exposure period (in days)	5
Number of shifts/periods with exposure in a week (1 to 7)	5
Observation settings	
Time of start of observation (time in hours)	0
Time of end of observation (time in hours)	88
Number of steps per hour	1000
Report times per hour	1

Table S2. Age, length of employment at current workplace and tobacco habits among the study participants.

		Workers	Controls
**Age (years)**	<25	-	-
	25-54	13	3
	55-64	-	1
**Employment (years)**	0-4	4	-
	5-9	2	-
	10+	5	4
**Smoking**	Never smoker	9	3
	Former smoker	2	1
	Smoker	2	-
**Snus***	Never snus	8	3
	Use snus	5	1

*Oral smokeless tobacco product placed behind the upper lip.

Table S3. Use of personal protective equipment (PPE).

		N
Used regular work gloves when working with creosote, this week?	Yes	10
	No	-
	Data not available	7
Used protective gloves when working with creosote, this week?	Yes	6
	No	1
	Data missing	10
How often do you change gloves when working with creosote?	Daily	12
	Once per week	1
	Once per month	-
	Data missing	4
Use of mask	Yes	2
	Yes, at special tasks	4
	No	7
	Data missing	4
Type of work clothes this week?	Long trousers / sweater	10
	T-shirt or shorts	3
	T-shirt and shorts	-
	Data missing	4
How often do you change work clothes?	Daily	-
	Every other day	1
	Once per week	11
	Data missing	5

Table S4. Mean, median, min and max concentration of pyrene (ng/cm^2) on different skin areas before and after a work shift.

Exposure class			N	Mean	Median	Min	Max
Exposed							
	Pre shift	Palm	13	2.6	2.3	0.33	9.5
	Post shift	Palm	13	33	21	0.31	140
		Back of hand	13	22	8.5	0.80	140
		Forehead	13	15	6.1	1.1	58
		Outside of calf	13	32	27	0.80	100
		Inside of calf	13	20	16	1.8	50
Controls							
	Pre shift	Palm	1	2.7	2.7	2.7	2.7
	Post shift	Palm	4	2.2	1.1	<0.23	6.5
		Back of hand	4	1.3	0.76	<0.23	3.4
		Forehead	3	3.2	0.12	<0.23	9.3
		Outside of calf	4	0.52	0.49	<0.23	1.0
		Inside of calf	4	0.53	0.46	<0.23	1.1

Table S5. Individual concentrations (ng/cm^2) of pyrene on different skin surfaces before and after a work shift. Samples were collected from exposed workers (*n*=13) and non-exposed controls (*n*=4).

Individuals	Pre shift	Post shift				
	Palm	Palm	Back of hand	Forehead	Outside of calf	Inside of calf
**Exposed workers**						
A*	0.61	1.4	0.80	2.3	1.3	1.8
B	2.5	7.3	8.5	6.1	8.5	7.7
C	0.49	21	4.9	58	41	28
D	4.0	38	4.1	3.8	100	50
E	2.3	13	26	23	27	46
F	3.1	32	25	18	46	16
G	0.33	0.93	4.7	1.1	11	5.5
H	3.2	140	49	26	28	17
I	9.5	54	140	4.7	55	26
J	1.9	33	10	37	0.80	14
K	4.0	75	9.7	13	76	37
L	0.93	16	2.9	4.7	13	15
Q*	0.61	0.31	1.2	1.7	9.4	2.0
Mean	2.6	33	22	15	32	20
Median	2.3	21	8.5	6.1	27	16
**Controls**						
M	n.a.	<0.23	<0.23	<0.23	<0.23	<0.23
N	n.a.	<0.23	<0.23	<0.23	<0.23	<0.23
O	2.7	6.5	3.4	n/a	1.0	1.1
P	n.a.	2.1	1.4	9.5	0.87	0.80
Mean	2.7	2.2	1.3	3.2	0.52	0.53
Median	2.7	1.1	0.76	0.12	0.49	0.46

*No documented contact with creosote-treated wood ties during the workday. n.a. = not available.

Table S6. Individual concentrations of 1-hydroxypyrene in urine (µmol/mol creatinine) for exposed workers and controls during the sampling week.

	Individuals	Monday Pre shift	Thursday Pre shift	Thursday Post shift
**Exposed workers**	A	1.4	2.2	1.9
	B[A]	3.0	9.6	8.7
	C	1.1	1.9	3.6
	D	1.1	2.9	2.7
	E	1.0	5.4	5.8
	F	3.5	16	12
	G	0.65	4.7	3.1
	H	3.3	12	11
	I	1.5	7.9	7.6
	J	1.5[B]	17	n.a.
	K	2.5	4.7 [B]	4.6
	L[A]	0.29	2.3 [B]	2.9
	Q	n.a.	n.a.	n.a.
Mean		1.7	7.2	5.8
Median		1.4	5.0	4.6
**Controls**	M	0.04	0.04	0.04
	N	0.04	0.04	0.03
	O	0.44	0.83	0.80
	P	0.34	0.78	0.88
Mean		0.22	0.42	0.44
Median		0.19	0.41	0.42

n.a.= not available. [A]No personal air measurements available. [B]Urine samples with creatinine concentrations exceeding the range of 0.3-3.0 g/L.

Figure S1. Certificates: German External Quality Assessment Scheme.

German External Quality Assessment Scheme

Intercomparison programme 57, 2016

for toxicological analyses in biological materials

Prof. Dr. med. H. Drexler

on behalf of the German Society for Occupational and Environmental Medicine .e.V

Schillerstr 25, D-91054 Erlangen

External Quality Control acc. to the Guidelines of the German Federal Medical Council

Teilnehmer: Environmental Analysis Unit 336
Occupational and Environmental Medicine
Att: Thomas Lundh, Dr Med Sci , Skånes University Hospital
22185 Lund
Sweden

Certificate

valid until July 31, 2017

This is to certify you participated in the intercomparison programme 57 / 2016 for occupational / environmental medical - toxicological analyses. In accordance with the guidelines issued by the German Federal Medical Council (Bundesärztekammer) of January 16th, 1987 and October 16th, 1987 and August 24th, 2001 on implementation of intercomparison programmes in the medical field you have fullfilled the requirements for the following parameters:

Environmental medical field

Pb in blood
3-PBA in urine
MEHP in urine
MBzP in urine
Trichloropyridinol in urine
Sn in urine
Pt in urine
Cotinine in urine
PFOA in serum
Cu in urine
Sb in urine
1-HP in urine
5-OH-MEHP in urine
PFOS in serum
Sr in urine
Mo in urine

Erlangen, 2016/06/24

Prof. Dr. med. H. Drexler

Prof. Dr. rer. nat. Th. Göen

German External Quality Assessment Scheme

Intercomparison programme 60, 2017
for toxicological analyses in biological materials

Prof. Dr. med. H. Drexler

on behalf of the German Society for Occupational and Environmental Medicine .e.V

Schillerstr 25, D-91054 Erlangen

External Quality Control acc. to the Guidelines of the German Federal Medical Council

Teilnehmer: Environmental Analysis Unit 336
Occupational and Environmental Medicine
Att: Thomas Lundh, Dr Med Sci
22185 Lund
Sweden

Certificate

valid until January 31, 2019

This is to certify you participated in the intercomparison programme 60 / 2017 for occupational / environmental medical - toxicological analyses. In accordance with the guidelines issued by the German Federal Medical Council (Bundesärztekammer) of January 16th, 1987 and October 16th, 1987 and August 24th, 2001 on implementation of intercomparison programmes in the medical field you have fullfilled the requirements for the following parameters:

Environmental medical field

Pb in blood	Hg in urine	Pt in urine
1-HP in urine	3-PBA in urine	Cotinine in urine
5-OH-MEHP in urine	5-carboxy-MEPP in urine	MEHP in urine
PFOS in serum	MnBP in urine	Cu in urine
Sr in urine	Zn in urine	Sb in urine
Mo in urine	Sn in urine	TCS in urine

Erlangen, 2018/01/08

Prof. Dr. med. H. Drexler Prof. Dr. rer. nat. Th. Göen

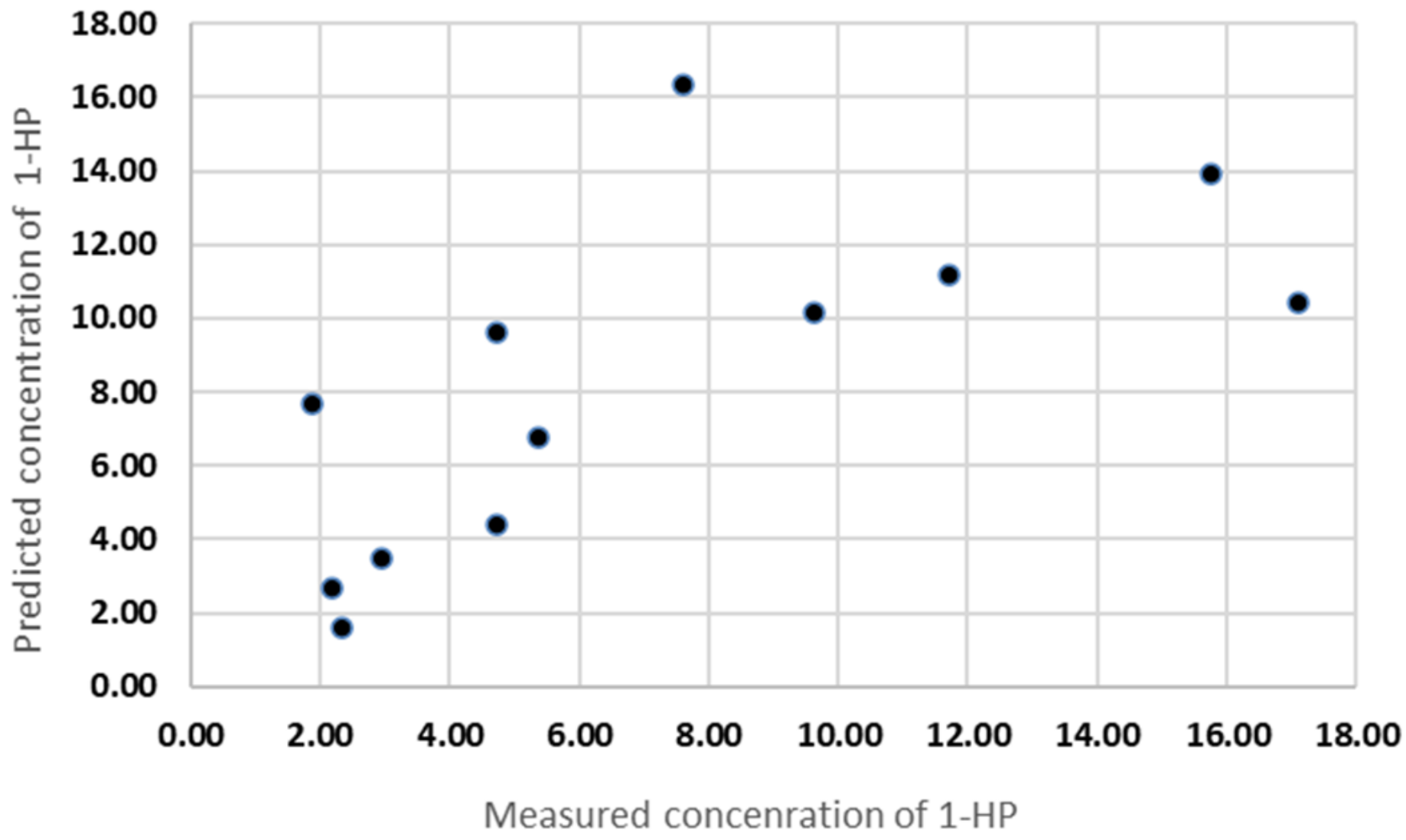

Figure S2. Scatter plot of measured and predicted urinary concentrations of 1-hydroxypyrene (1-HP) for urine samples collected before the work shift on Thursday. All concentrations are presented in µmol/mol creatinine. Urine 1-HP levels are normalised to creatinine for the corresponding samples. The skin surface area used for modelling dermal uptake was 1000 cm^2.

References

Alhamdow A, Lindh C, Albin M, Gustavsson P, Tinnerberg H, Broberg K. Early markers of cardiovascular disease are associated with occupational exposure to polycyclic aromatic hydrocarbons. Sci Rep. 2017:7:9426-. doi: 10.1038/s41598-017-09956-x.

Bramming Jørgensen R, Strandberg B, Sjaastad AK, Johansen A, Svendsen K. Simulated restaurant cook exposure to emissions of PAHs, mutagenic aldehydes, and particles from frying bacon. J Occup Environ Hyg. 2013:10:122-131. doi: 10.1080/15459624.2012.755864.

Kammer R, Tinnerberg H, Eriksson K. Evaluation of a tape-stripping technique for measuring dermal exposure to pyrene and benzo(a)pyrene. J Environ Monit. 2011:13:2165–2171. doi: 10.1039/c1em10245a.
